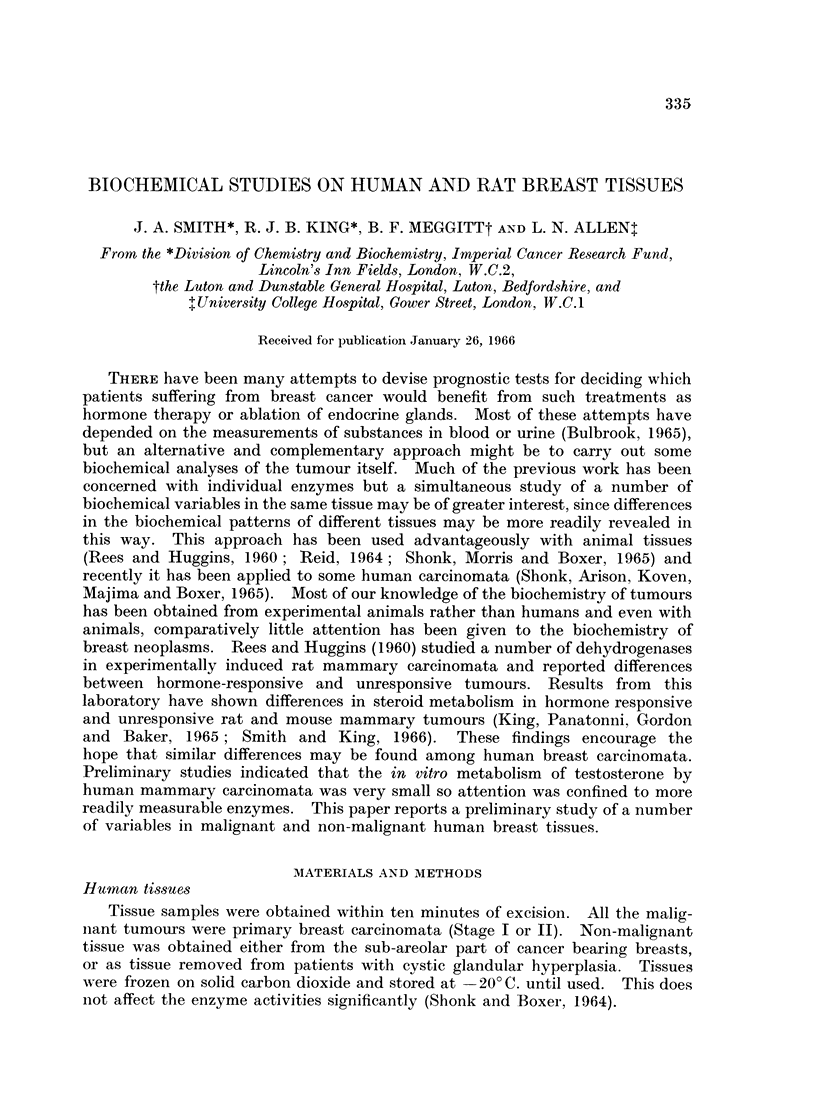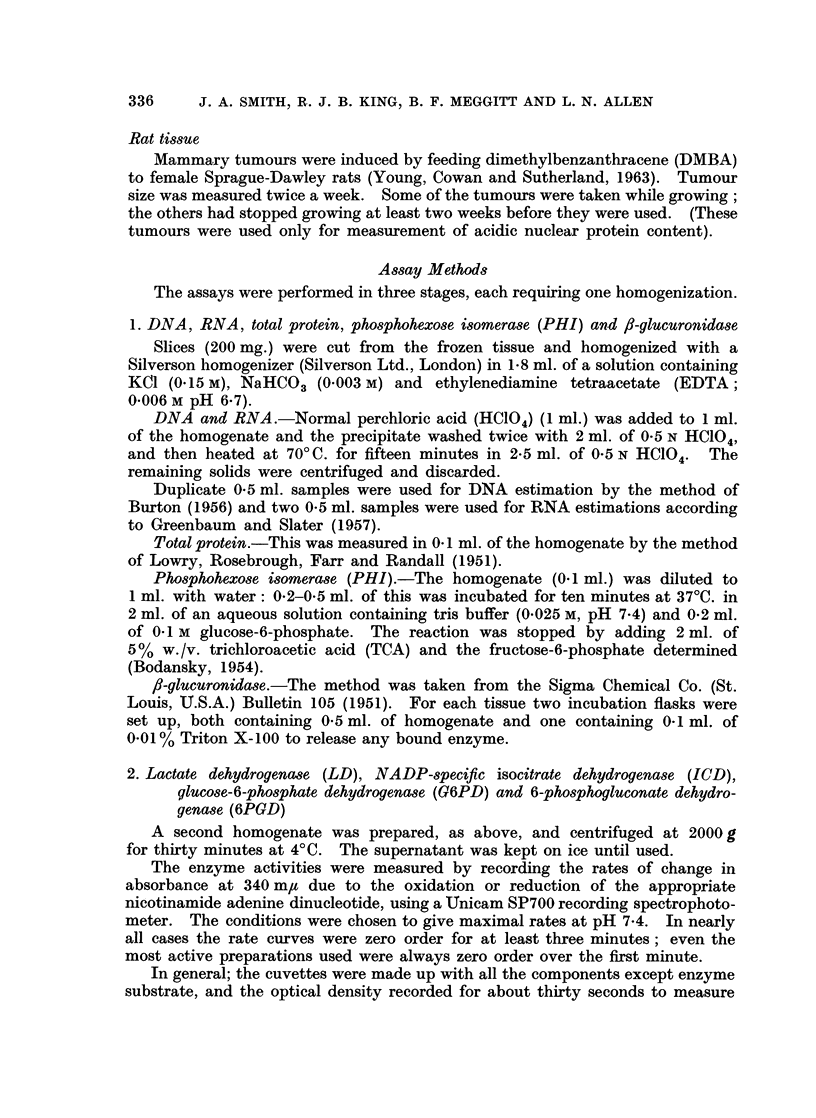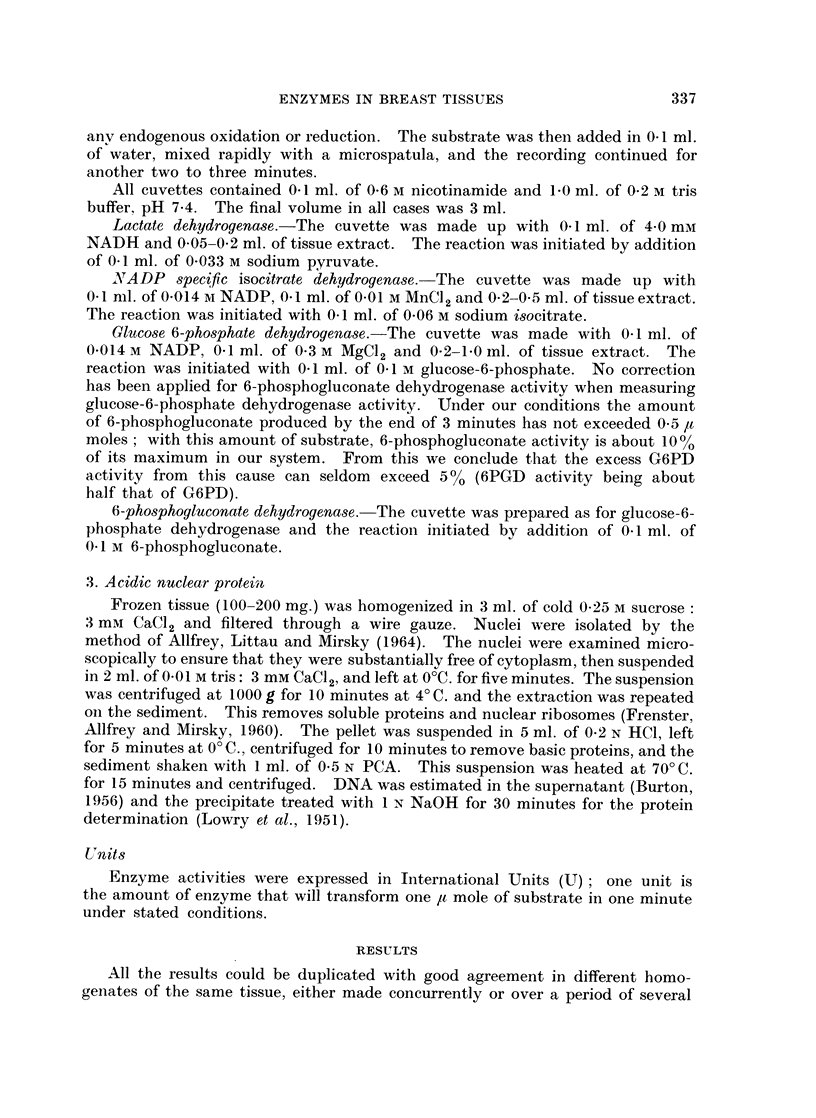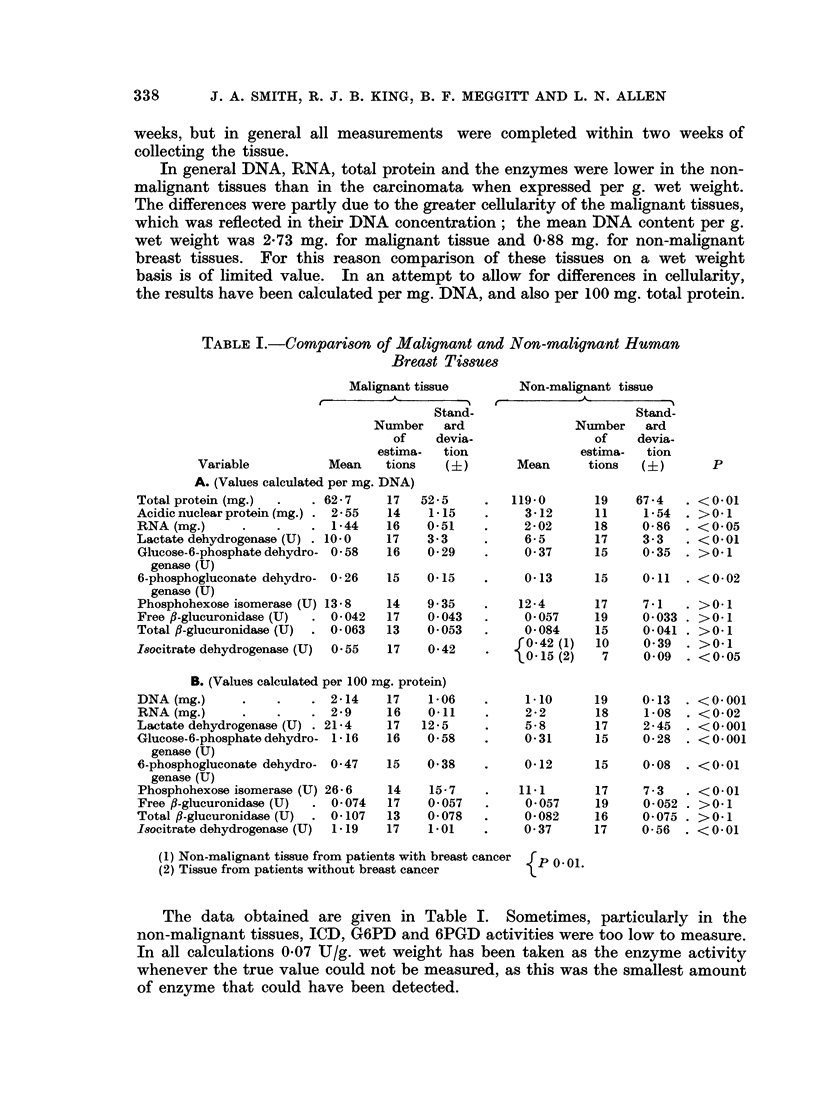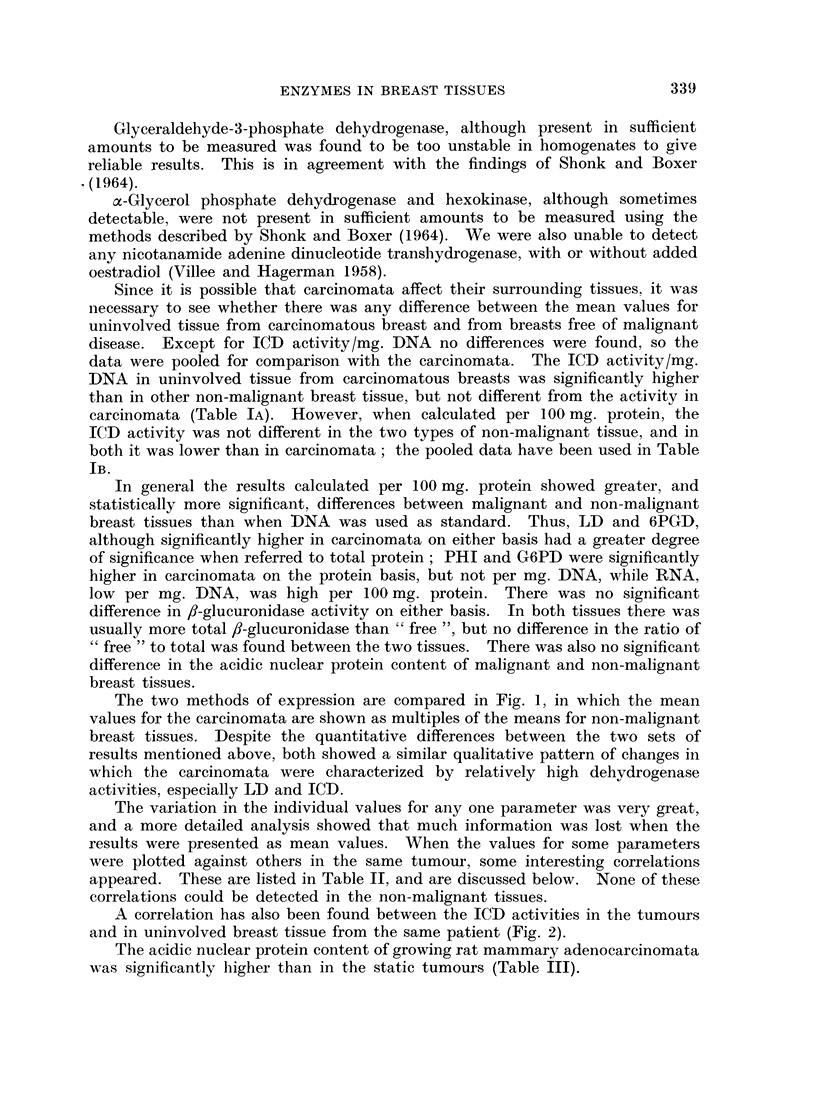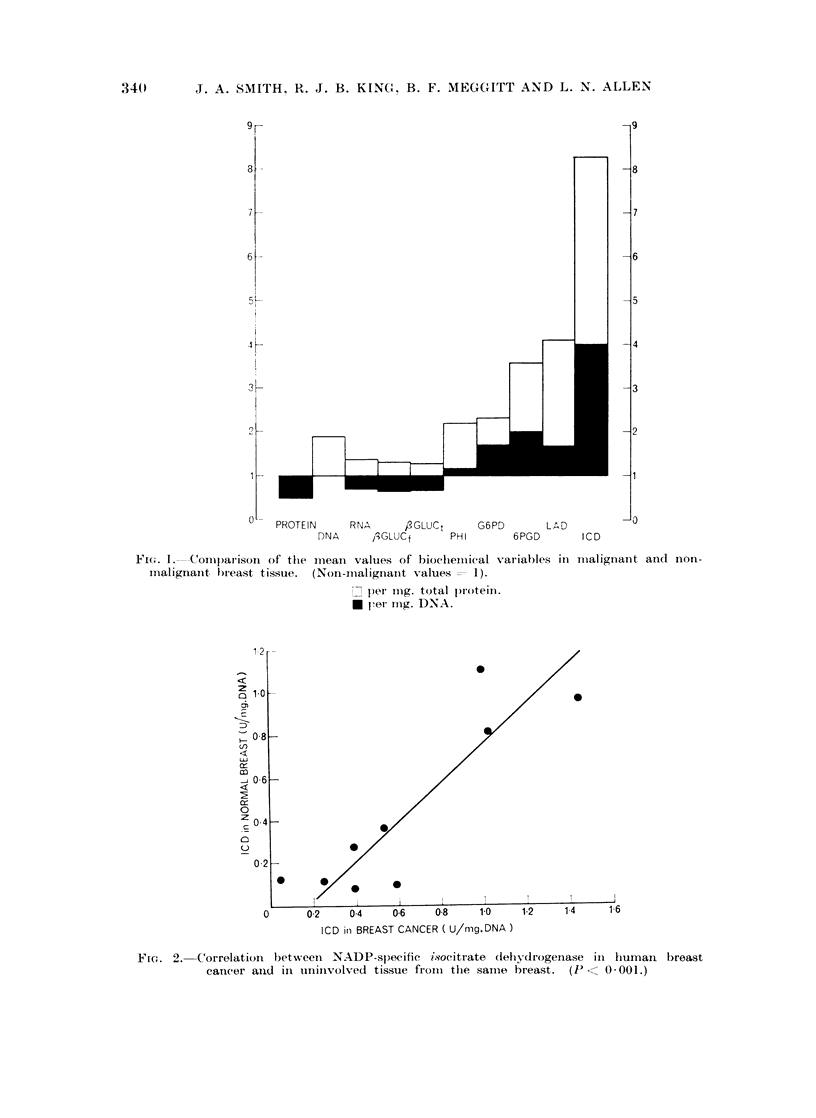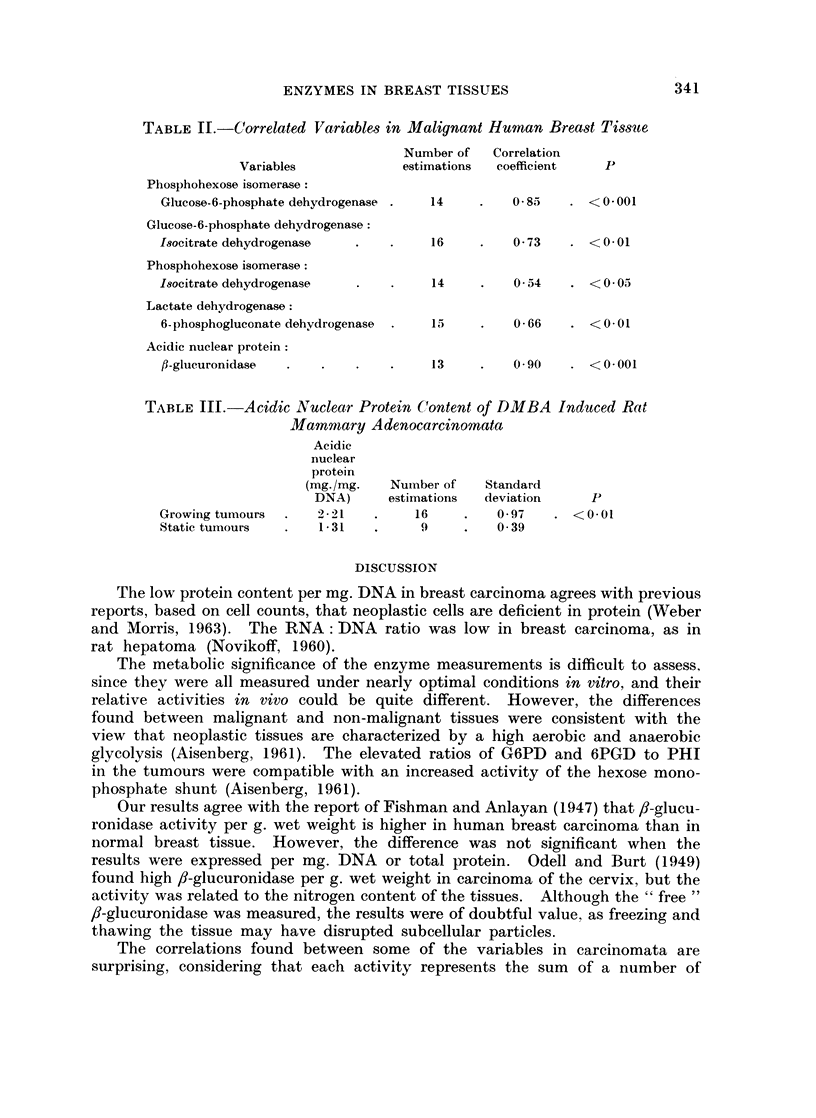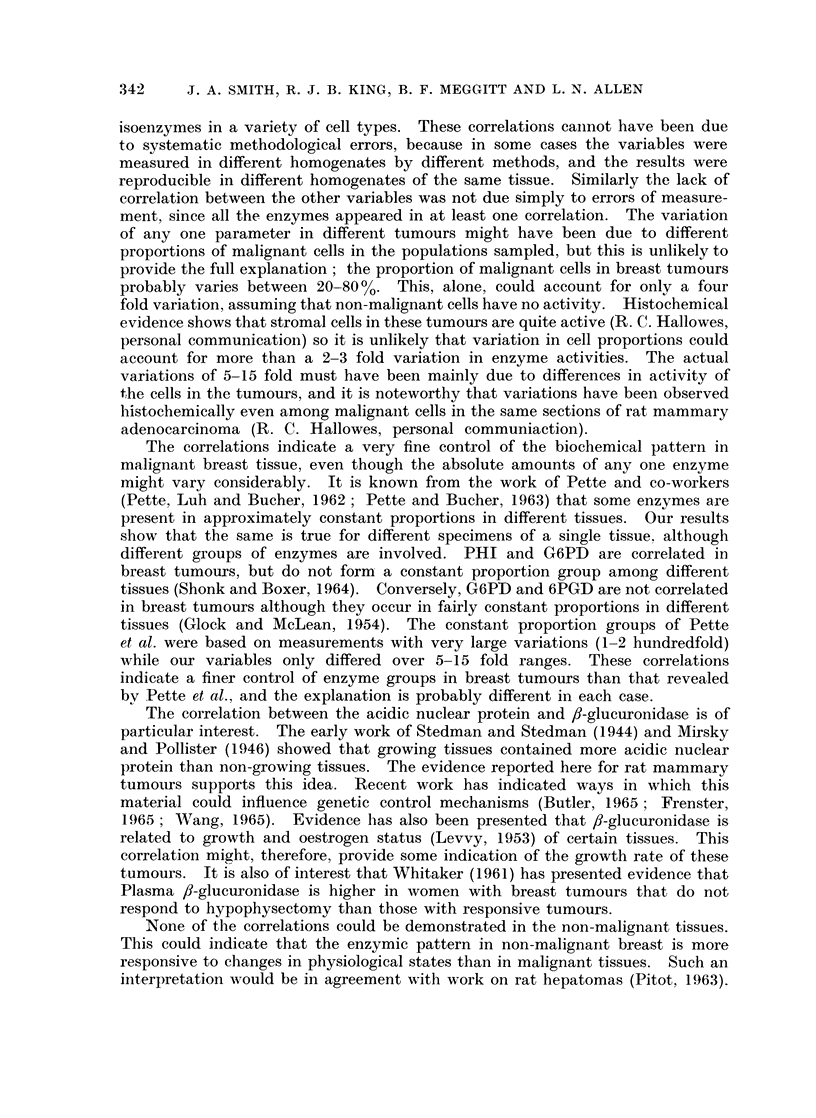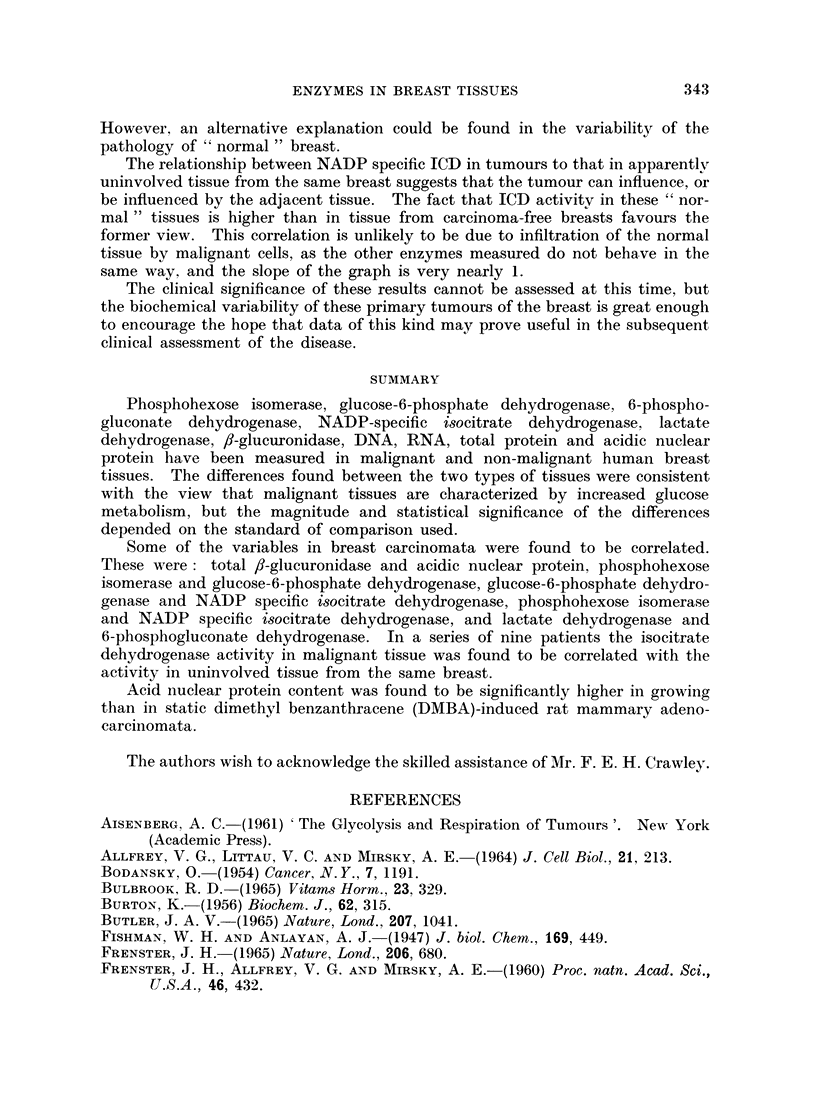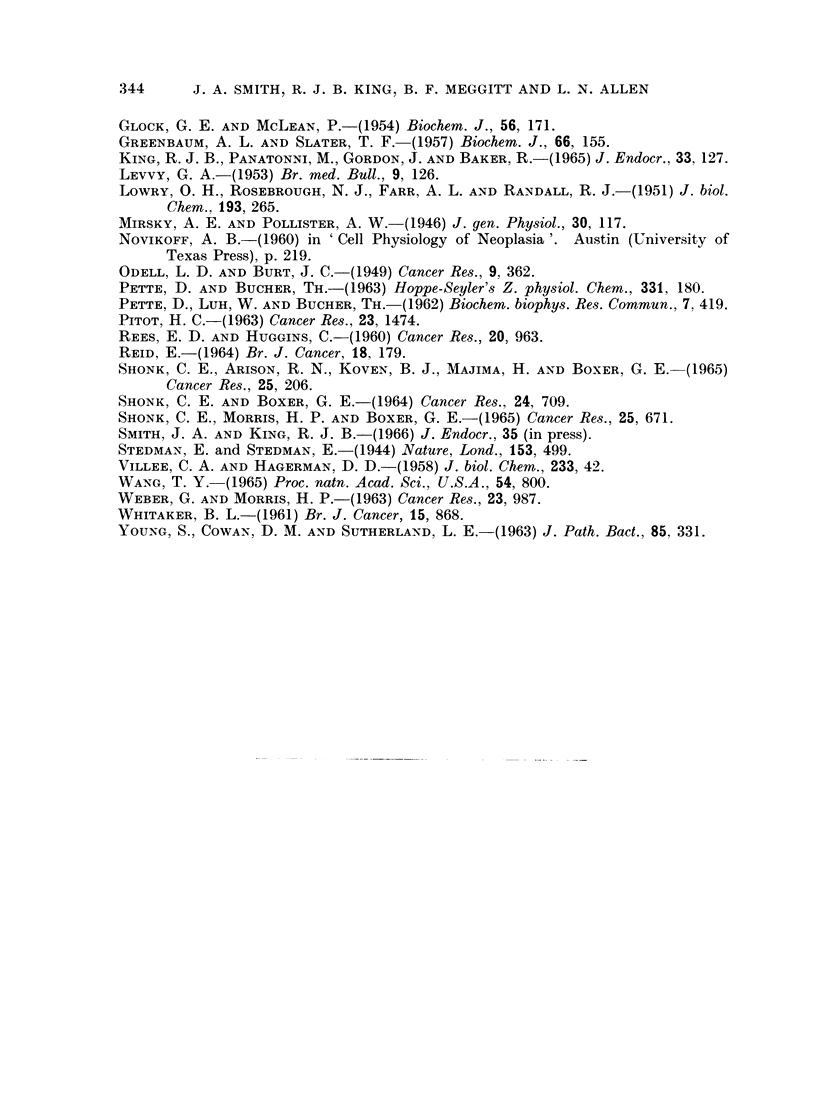# Biochemical studies on human and rat breast tissues.

**DOI:** 10.1038/bjc.1966.41

**Published:** 1966-06

**Authors:** J. A. Smith, R. J. King, B. F. Meggitt, L. N. Allen


### BIOCHEMICAL STUDIES ON HUMAN AND RAT BREAST TISSUES

J. A. SMITH*, R. J. B. KING*, B. F. MEGGITT† AND L. N. ALLEN‡

*From the *Division of Chemistry and Biochemistry, Imperial Cancer Research Fund, Lincoln's Inn Fields, London, W.C.2,*
*†the Luton and Dunstable General Hospital, Luton, Bedfordshire, and*
*‡University College Hospital, Gower Street, London, W.C.1*

Received for publication January 26, 1966

THERE have been many attempts to devise prognostic tests for deciding which patients suffering from breast cancer would benefit from such treatments as hormone therapy or ablation of endocrine glands. Most of these attempts have depended on the measurements of substances in blood or urine (Bulbrook, 1965), but an alternative and complementary approach might be to carry out some biochemical analyses of the tumour itself. Much of the previous work has been concerned with individual enzymes but a simultaneous study of a number of biochemical variables in the same tissue may be of greater interest, since differences in the biochemical patterns of different tissues may be more readily revealed in this way. This approach has been used advantageously with animal tissues (Rees and Huggins, 1960; Reid, 1964; Shonk, Morris and Boxer, 1965) and recently it has been applied to some human carcinomata (Shonk, Arison, Koven, Majima and Boxer, 1965). Most of our knowledge of the biochemistry of tumours has been obtained from experimental animals rather than humans and even with animals, comparatively little attention has been given to the biochemistry of breast neoplasms. Rees and Huggins (1960) studied a number of dehydrogenases in experimentally induced rat mammary carcinomata and reported differences between hormone-responsive and unresponsive tumours. Results from this laboratory have shown differences in steroid metabolism in hormone responsive and unresponsive rat and mouse mammary tumours (King, Panatonni, Gordon and Baker, 1965; Smith and King, 1966). These findings encourage the hope that similar differences may be found among human breast carcinomata. Preliminary studies indicated that the *in vitro* metabolism of testosterone by human mammary carcinomata was very small so attention was confined to more readily measurable enzymes. This paper reports a preliminary study of a number of variables in malignant and non-malignant human breast tissues.

### MATERIALS AND METHODS

### *Human tissues*

Tissue samples were obtained within ten minutes of excision. All the malignant tumours were primary breast carcinomata (Stage I or II). Non-malignant tissue was obtained either from the sub-areolar part of cancer bearing breasts, or as tissue removed from patients with cystic glandular hyperplasia. Tissues were frozen on solid carbon dioxide and stored at $-20°$ C. until used. This does not affect the enzyme activities significantly (Shonk and Boxer, 1964).

*Rat tissue*

Mammary tumours were induced by feeding dimethylbenzanthracene (DMBA) to female Sprague-Dawley rats (Young, Cowan and Sutherland, 1963). Tumour size was measured twice a week. Some of the tumours were taken while growing ; the others had stopped growing at least two weeks before they were used. (These tumours were used only for measurement of acidic nuclear protein content).

### *Assay Methods*

The assays were performed in three stages, each requiring one homogenization.

1. *DNA, RNA, total protein, phosphohexose isomerase (PHI) and β-glucuronidase*

Slices (200 mg.) were cut from the frozen tissue and homogenized with a Silverson homogenizer (Silverson Ltd., London) in 1·8 ml. of a solution containing KCl (0·15 M), $NaHCO_3$ (0·003 M) and ethylenediamine tetraacetate (EDTA ; 0·006 M pH 6·7).

*DNA and RNA.*—Normal perchloric acid ($HClO_4$) (1 ml.) was added to 1 ml. of the homogenate and the precipitate washed twice with 2 ml. of 0·5 N $HClO_4$, and then heated at 70° C. for fifteen minutes in 2·5 ml. of 0·5 N $HClO_4$. The remaining solids were centrifuged and discarded.

Duplicate 0·5 ml. samples were used for DNA estimation by the method of Burton (1956) and two 0·5 ml. samples were used for RNA estimations according to Greenbaum and Slater (1957).

*Total protein.*—This was measured in 0·1 ml. of the homogenate by the method of Lowry, Rosebrough, Farr and Randall (1951).

*Phosphohexose isomerase (PHI).*—The homogenate (0·1 ml.) was diluted to 1 ml. with water : 0·2–0·5 ml. of this was incubated for ten minutes at 37°C. in 2 ml. of an aqueous solution containing tris buffer (0·025 M, pH 7·4) and 0·2 ml. of 0·1 M glucose-6-phosphate. The reaction was stopped by adding 2 ml. of 5% w./v. trichloroacetic acid (TCA) and the fructose-6-phosphate determined (Bodansky, 1954).

*β-glucuronidase.*—The method was taken from the Sigma Chemical Co. (St. Louis, U.S.A.) Bulletin 105 (1951). For each tissue two incubation flasks were set up, both containing 0·5 ml. of homogenate and one containing 0·1 ml. of 0·01% Triton X-100 to release any bound enzyme.

2. *Lactate dehydrogenase (LD), NADP-specific* isocitrate *dehydrogenase (ICD), glucose-6-phosphate dehydrogenase (G6PD) and 6-phosphogluconate dehydrogenase (6PGD)*

A second homogenate was prepared, as above, and centrifuged at 2000 **g** for thirty minutes at 4°C. The supernatant was kept on ice until used.

The enzyme activities were measured by recording the rates of change in absorbance at 340 mμ due to the oxidation or reduction of the appropriate nicotinamide adenine dinucleotide, using a Unicam SP700 recording spectrophotometer. The conditions were chosen to give maximal rates at pH 7·4. In nearly all cases the rate curves were zero order for at least three minutes ; even the most active preparations used were always zero order over the first minute.

In general; the cuvettes were made up with all the components except enzyme substrate, and the optical density recorded for about thirty seconds to measure any endogenous oxidation or reduction. The substrate was then added in 0·1 ml. of water, mixed rapidly with a microspatula, and the recording continued for another two to three minutes.

All cuvettes contained 0·1 ml. of 0·6 M nicotinamide and 1·0 ml. of 0·2 M tris buffer, pH 7·4. The final volume in all cases was 3 ml.

*Lactate dehydrogenase.*—The cuvette was made up with 0·1 ml. of 4·0 mM NADH and 0·05–0·2 ml. of tissue extract. The reaction was initiated by addition of 0·1 ml. of 0·033 M sodium pyruvate.

*NADP specific* iso*citrate dehydrogenase.*—The cuvette was made up with 0·1 ml. of 0·014 M NADP, 0·1 ml. of 0·01 M $MnCl_2$ and 0·2–0·5 ml. of tissue extract. The reaction was initiated with 0·1 ml. of 0·06 M sodium *iso*citrate.

*Glucose 6-phosphate dehydrogenase.*—The cuvette was made with 0·1 ml. of 0·014 M NADP, 0·1 ml. of 0·3 M $MgCl_2$ and 0·2–1·0 ml. of tissue extract. The reaction was initiated with 0·1 ml. of 0·1 M glucose-6-phosphate. No correction has been applied for 6-phosphogluconate dehydrogenase activity when measuring glucose-6-phosphate dehydrogenase activity. Under our conditions the amount of 6-phosphogluconate produced by the end of 3 minutes has not exceeded 0·5 $\mu$ moles ; with this amount of substrate, 6-phosphogluconate activity is about 10% of its maximum in our system. From this we conclude that the excess G6PD activity from this cause can seldom exceed 5% (6PGD activity being about half that of G6PD).

*6-phosphogluconate dehydrogenase.*—The cuvette was prepared as for glucose-6-phosphate dehydrogenase and the reaction initiated by addition of 0·1 ml. of 0·1 M 6-phosphogluconate.

3. *Acidic nuclear protein*

Frozen tissue (100–200 mg.) was homogenized in 3 ml. of cold 0·25 M sucrose : 3 mM $CaCl_2$ and filtered through a wire gauze. Nuclei were isolated by the method of Allfrey, Littau and Mirsky (1964). The nuclei were examined microscopically to ensure that they were substantially free of cytoplasm, then suspended in 2 ml. of 0·01 M tris : 3 mM $CaCl_2$, and left at 0°C. for five minutes. The suspension was centrifuged at 1000 *g* for 10 minutes at 4° C. and the extraction was repeated on the sediment. This removes soluble proteins and nuclear ribosomes (Frenster, Allfrey and Mirsky, 1960). The pellet was suspended in 5 ml. of 0·2 N HCl, left for 5 minutes at 0° C., centrifuged for 10 minutes to remove basic proteins, and the sediment shaken with 1 ml. of 0·5 N PCA. This suspension was heated at 70° C. for 15 minutes and centrifuged. DNA was estimated in the supernatant (Burton, 1956) and the precipitate treated with 1 N NaOH for 30 minutes for the protein determination (Lowry *et al.*, 1951).

*Units*

Enzyme activities were expressed in International Units (U) ; one unit is the amount of enzyme that will transform one $\mu$ mole of substrate in one minute under stated conditions.

### RESULTS

All the results could be duplicated with good agreement in different homogenates of the same tissue, either made concurrently or over a period of several weeks, but in general all measurements were completed within two weeks of collecting the tissue.

In general DNA, RNA, total protein and the enzymes were lower in the non-malignant tissues than in the carcinomata when expressed per g. wet weight. The differences were partly due to the greater cellularity of the malignant tissues, which was reflected in their DNA concentration ; the mean DNA content per g. wet weight was 2·73 mg. for malignant tissue and 0·88 mg. for non-malignant breast tissues. For this reason comparison of these tissues on a wet weight basis is of limited value. In an attempt to allow for differences in cellularity, the results have been calculated per mg. DNA, and also per 100 mg. total protein.

TABLE I.—*Comparison of Malignant and Non-malignant Human Breast Tissues*

| | Malignant tissue | | | Non-malignant tissue | | | |
|---|---|---|---|---|---|---|---|
| Variable | Mean | Number of estimations | Standard deviation (±) | Mean | Number of estimations | Standard deviation (±) | *P* |
| **A.** (Values calculated per mg. DNA) | | | | | | | |
| Total protein (mg.) | 62·7 | 17 | 52·5 | 119·0 | 19 | 67·4 | <0·01 |
| Acidic nuclear protein (mg.) | 2·55 | 14 | 1·15 | 3·12 | 11 | 1·54 | >0·1 |
| RNA (mg.) | 1·44 | 16 | 0·51 | 2·02 | 18 | 0·86 | <0·05 |
| Lactate dehydrogenase (U) | 10·0 | 17 | 3·3 | 6·5 | 17 | 3·3 | <0·01 |
| Glucose-6-phosphate dehydrogenase (U) | 0·58 | 16 | 0·29 | 0·37 | 15 | 0·35 | >0·1 |
| 6-phosphogluconate dehydrogenase (U) | 0·26 | 15 | 0·15 | 0·13 | 15 | 0·11 | <0·02 |
| Phosphohexose isomerase (U) | 13·8 | 14 | 9·35 | 12·4 | 17 | 7·1 | >0·1 |
| Free β-glucuronidase (U) | 0·042 | 17 | 0·043 | 0·057 | 19 | 0·033 | >0·1 |
| Total β-glucuronidase (U) | 0·063 | 13 | 0·053 | 0·084 | 15 | 0·041 | >0·1 |
| *Iso*citrate dehydrogenase (U) | 0·55 | 17 | 0·42 | {0·42 (1) | 10 | 0·39 | >0·1 |
| | | | | {0·15 (2) | 7 | 0·09 | <0·05 |
| **B.** (Values calculated per 100 mg. protein) | | | | | | | |
| DNA (mg.) | 2·14 | 17 | 1·06 | 1·10 | 19 | 0·13 | <0·001 |
| RNA (mg.) | 2·9 | 16 | 0·11 | 2·2 | 18 | 1·08 | <0·02 |
| Lactate dehydrogenase (U) | 21·4 | 17 | 12·5 | 5·8 | 17 | 2·45 | <0·001 |
| Glucose-6-phosphate dehydrogenase (U) | 1·16 | 16 | 0·58 | 0·31 | 15 | 0·28 | <0·001 |
| 6-phosphogluconate dehydrogenase (U) | 0·47 | 15 | 0·38 | 0·12 | 15 | 0·08 | <0·01 |
| Phosphohexose isomerase (U) | 26·6 | 14 | 15·7 | 11·1 | 17 | 7·3 | <0·01 |
| Free β-glucuronidase (U) | 0·074 | 17 | 0·057 | 0·057 | 19 | 0·052 | >0·1 |
| Total β-glucuronidase (U) | 0·107 | 13 | 0·078 | 0·082 | 16 | 0·075 | >0·1 |
| *Iso*citrate dehydrogenase (U) | 1·19 | 17 | 1·01 | 0·37 | 17 | 0·56 | <0·01 |

(1) Non-malignant tissue from patients with breast cancer
(2) Tissue from patients without breast cancer
} *P* 0·01.

The data obtained are given in Table I. Sometimes, particularly in the non-malignant tissues, ICD, G6PD and 6PGD activities were too low to measure. In all calculations 0·07 U/g. wet weight has been taken as the enzyme activity whenever the true value could not be measured, as this was the smallest amount of enzyme that could have been detected.

Glyceraldehyde-3-phosphate dehydrogenase, although present in sufficient amounts to be measured was found to be too unstable in homogenates to give reliable results. This is in agreement with the findings of Shonk and Boxer (1964).

α-Glycerol phosphate dehydrogenase and hexokinase, although sometimes detectable, were not present in sufficient amounts to be measured using the methods described by Shonk and Boxer (1964). We were also unable to detect any nicotanamide adenine dinucleotide transhydrogenase, with or without added oestradiol (Villee and Hagerman 1958).

Since it is possible that carcinomata affect their surrounding tissues, it was necessary to see whether there was any difference between the mean values for uninvolved tissue from carcinomatous breast and from breasts free of malignant disease. Except for ICD activity/mg. DNA no differences were found, so the data were pooled for comparison with the carcinomata. The ICD activity/mg. DNA in uninvolved tissue from carcinomatous breasts was significantly higher than in other non-malignant breast tissue, but not different from the activity in carcinomata (Table IA). However, when calculated per 100 mg. protein, the ICD activity was not different in the two types of non-malignant tissue, and in both it was lower than in carcinomata ; the pooled data have been used in Table IB.

In general the results calculated per 100 mg. protein showed greater, and statistically more significant, differences between malignant and non-malignant breast tissues than when DNA was used as standard. Thus, LD and 6PGD, although significantly higher in carcinomata on either basis had a greater degree of significance when referred to total protein ; PHI and G6PD were significantly higher in carcinomata on the protein basis, but not per mg. DNA, while RNA, low per mg. DNA, was high per 100 mg. protein. There was no significant difference in β-glucuronidase activity on either basis. In both tissues there was usually more total β-glucuronidase than " free ", but no difference in the ratio of " free " to total was found between the two tissues. There was also no significant difference in the acidic nuclear protein content of malignant and non-malignant breast tissues.

The two methods of expression are compared in Fig. 1, in which the mean values for the carcinomata are shown as multiples of the means for non-malignant breast tissues. Despite the quantitative differences between the two sets of results mentioned above, both showed a similar qualitative pattern of changes in which the carcinomata were characterized by relatively high dehydrogenase activities, especially LD and ICD.

The variation in the individual values for any one parameter was very great, and a more detailed analysis showed that much information was lost when the results were presented as mean values. When the values for some parameters were plotted against others in the same tumour, some interesting correlations appeared. These are listed in Table II, and are discussed below. None of these correlations could be detected in the non-malignant tissues.

A correlation has also been found between the ICD activities in the tumours and in uninvolved breast tissue from the same patient (Fig. 2).

The acidic nuclear protein content of growing rat mammary adenocarcinomata was significantly higher than in the static tumours (Table III).

FIG. 1.—Comparison of the mean values of biochemical variables in malignant and non-malignant breast tissue. (Non-malignant values = 1).

□ per mg. total protein.

■ per mg. DNA.

FIG. 2.—Correlation between NADP-specific *iso*citrate dehydrogenase in human breast cancer and in uninvolved tissue from the same breast. ($P < 0 \cdot 001$.)

TABLE II.—*Correlated Variables in Malignant Human Breast Tissue*

| Variables | Number of estimations | Correlation coefficient | *P* |
|---|---|---|---|
| Phosphohexose isomerase : | | | |
| Glucose-6-phosphate dehydrogenase | 14 | 0·85 | <0·001 |
| Glucose-6-phosphate dehydrogenase : | | | |
| *Iso*citrate dehydrogenase | 16 | 0·73 | <0·01 |
| Phosphohexose isomerase : | | | |
| *Iso*citrate dehydrogenase | 14 | 0·54 | <0·05 |
| Lactate dehydrogenase : | | | |
| 6-phosphogluconate dehydrogenase | 15 | 0·66 | <0·01 |
| Acidic nuclear protein : | | | |
| β-glucuronidase | 13 | 0·90 | <0·001 |

TABLE III.—*Acidic Nuclear Protein Content of DMBA Induced Rat Mammary Adenocarcinomata*

| | Acidic nuclear protein (mg./mg. DNA) | Number of estimations | Standard deviation | *P* |
|---|---|---|---|---|
| Growing tumours | 2·21 | 16 | 0·97 | <0·01 |
| Static tumours | 1·31 | 9 | 0·39 | |

### DISCUSSION

The low protein content per mg. DNA in breast carcinoma agrees with previous reports, based on cell counts, that neoplastic cells are deficient in protein (Weber and Morris, 1963). The RNA : DNA ratio was low in breast carcinoma, as in rat hepatoma (Novikoff, 1960).

The metabolic significance of the enzyme measurements is difficult to assess, since they were all measured under nearly optimal conditions *in vitro*, and their relative activities *in vivo* could be quite different. However, the differences found between malignant and non-malignant tissues were consistent with the view that neoplastic tissues are characterized by a high aerobic and anaerobic glycolysis (Aisenberg, 1961). The elevated ratios of G6PD and 6PGD to PHI in the tumours were compatible with an increased activity of the hexose monophosphate shunt (Aisenberg, 1961).

Our results agree with the report of Fishman and Anlayan (1947) that β-glucuronidase activity per g. wet weight is higher in human breast carcinoma than in normal breast tissue. However, the difference was not significant when the results were expressed per mg. DNA or total protein. Odell and Burt (1949) found high β-glucuronidase per g. wet weight in carcinoma of the cervix, but the activity was related to the nitrogen content of the tissues. Although the " free " β-glucuronidase was measured, the results were of doubtful value, as freezing and thawing the tissue may have disrupted subcellular particles.

The correlations found between some of the variables in carcinomata are surprising, considering that each activity represents the sum of a number of isoenzymes in a variety of cell types. These correlations cannot have been due to systematic methodological errors, because in some cases the variables were measured in different homogenates by different methods, and the results were reproducible in different homogenates of the same tissue. Similarly the lack of correlation between the other variables was not due simply to errors of measurement, since all the enzymes appeared in at least one correlation. The variation of any one parameter in different tumours might have been due to different proportions of malignant cells in the populations sampled, but this is unlikely to provide the full explanation ; the proportion of malignant cells in breast tumours probably varies between 20–80%. This, alone, could account for only a four fold variation, assuming that non-malignant cells have no activity. Histochemical evidence shows that stromal cells in these tumours are quite active (R. C. Hallowes, personal communication) so it is unlikely that variation in cell proportions could account for more than a 2–3 fold variation in enzyme activities. The actual variations of 5–15 fold must have been mainly due to differences in activity of the cells in the tumours, and it is noteworthy that variations have been observed histochemically even among malignant cells in the same sections of rat mammary adenocarcinoma (R. C. Hallowes, personal communiaction).

The correlations indicate a very fine control of the biochemical pattern in malignant breast tissue, even though the absolute amounts of any one enzyme might vary considerably. It is known from the work of Pette and co-workers (Pette, Luh and Bucher, 1962 ; Pette and Bucher, 1963) that some enzymes are present in approximately constant proportions in different tissues. Our results show that the same is true for different specimens of a single tissue, although different groups of enzymes are involved. PHI and G6PD are correlated in breast tumours, but do not form a constant proportion group among different tissues (Shonk and Boxer, 1964). Conversely, G6PD and 6PGD are not correlated in breast tumours although they occur in fairly constant proportions in different tissues (Glock and McLean, 1954). The constant proportion groups of Pette *et al.* were based on measurements with very large variations (1–2 hundredfold) while our variables only differed over 5–15 fold ranges. These correlations indicate a finer control of enzyme groups in breast tumours than that revealed by Pette *et al.*, and the explanation is probably different in each case.

The correlation between the acidic nuclear protein and $\beta$-glucuronidase is of particular interest. The early work of Stedman and Stedman (1944) and Mirsky and Pollister (1946) showed that growing tissues contained more acidic nuclear protein than non-growing tissues. The evidence reported here for rat mammary tumours supports this idea. Recent work has indicated ways in which this material could influence genetic control mechanisms (Butler, 1965 ; Frenster, 1965 ; Wang, 1965). Evidence has also been presented that $\beta$-glucuronidase is related to growth and oestrogen status (Levvy, 1953) of certain tissues. This correlation might, therefore, provide some indication of the growth rate of these tumours. It is also of interest that Whitaker (1961) has presented evidence that Plasma $\beta$-glucuronidase is higher in women with breast tumours that do not respond to hypophysectomy than those with responsive tumours.

None of the correlations could be demonstrated in the non-malignant tissues. This could indicate that the enzymic pattern in non-malignant breast is more responsive to changes in physiological states than in malignant tissues. Such an interpretation would be in agreement with work on rat hepatomas (Pitot, 1963).

However, an alternative explanation could be found in the variability of the pathology of " normal " breast.

The relationship between NADP specific ICD in tumours to that in apparently uninvolved tissue from the same breast suggests that the tumour can influence, or be influenced by the adjacent tissue. The fact that ICD activity in these " normal " tissues is higher than in tissue from carcinoma-free breasts favours the former view. This correlation is unlikely to be due to infiltration of the normal tissue by malignant cells, as the other enzymes measured do not behave in the same way, and the slope of the graph is very nearly 1.

The clinical significance of these results cannot be assessed at this time, but the biochemical variability of these primary tumours of the breast is great enough to encourage the hope that data of this kind may prove useful in the subsequent clinical assessment of the disease.

### SUMMARY

Phosphohexose isomerase, glucose-6-phosphate dehydrogenase, 6-phosphogluconate dehydrogenase, NADP-specific *iso*citrate dehydrogenase, lactate dehydrogenase, β-glucuronidase, DNA, RNA, total protein and acidic nuclear protein have been measured in malignant and non-malignant human breast tissues. The differences found between the two types of tissues were consistent with the view that malignant tissues are characterized by increased glucose metabolism, but the magnitude and statistical significance of the differences depended on the standard of comparison used.

Some of the variables in breast carcinomata were found to be correlated. These were : total β-glucuronidase and acidic nuclear protein, phosphohexose isomerase and glucose-6-phosphate dehydrogenase, glucose-6-phosphate dehydrogenase and NADP specific *iso*citrate dehydrogenase, phosphohexose isomerase and NADP specific *iso*citrate dehydrogenase, and lactate dehydrogenase and 6-phosphogluconate dehydrogenase. In a series of nine patients the isocitrate dehydrogenase activity in malignant tissue was found to be correlated with the activity in uninvolved tissue from the same breast.

Acid nuclear protein content was found to be significantly higher in growing than in static dimethyl benzanthracene (DMBA)-induced rat mammary adenocarcinomata.

The authors wish to acknowledge the skilled assistance of Mr. F. E. H. Crawley.

### REFERENCES

Aisenberg, A. C.—(1961) ' The Glycolysis and Respiration of Tumours '. New York (Academic Press).

Allfrey, V. G., Littau, V. C. and Mirsky, A. E.—(1964) *J. Cell Biol.*, **21**, 213.

Bodansky, O.—(1954) *Cancer, N.Y.*, **7**, 1191.

Bulbrook, R. D.—(1965) *Vitams Horm.*, **23**, 329.

Burton, K.—(1956) *Biochem. J.*, **62**, 315.

Butler, J. A. V.—(1965) *Nature, Lond.*, **207**, 1041.

Fishman, W. H. and Anlayan, A. J.—(1947) *J. biol. Chem.*, **169**, 449.

Frenster, J. H.—(1965) *Nature, Lond.*, **206**, 680.

Frenster, J. H., Allfrey, V. G. and Mirsky, A. E.—(1960) *Proc. natn. Acad. Sci., U.S.A.*, **46**, 432.

GLOCK, G. E. AND McLEAN, P.—(1954) *Biochem. J.*, **56**, 171.
GREENBAUM, A. L. AND SLATER, T. F.—(1957) *Biochem. J.*, **66**, 155.
KING, R. J. B., PANATONNI, M., GORDON, J. AND BAKER, R.—(1965) *J. Endocr.*, **33**, 127.
LEVVY, G. A.—(1953) *Br. med. Bull.*, **9**, 126.
LOWRY, O. H., ROSEBROUGH, N. J., FARR, A. L. AND RANDALL, R. J.—(1951) *J. biol. Chem.*, **193**, 265.
MIRSKY, A. E. AND POLLISTER, A. W.—(1946) *J. gen. Physiol.*, **30**, 117.
NOVIKOFF, A. B.—(1960) in ' Cell Physiology of Neoplasia '. Austin (University of Texas Press), p. 219.
ODELL, L. D. AND BURT, J. C.—(1949) *Cancer Res.*, **9**, 362.
PETTE, D. AND BUCHER, TH.—(1963) *Hoppe-Seyler's Z. physiol. Chem.*, **331**, 180.
PETTE, D., LUH, W. AND BUCHER, TH.—(1962) *Biochem. biophys. Res. Commun.*, **7**, 419.
PITOT, H. C.—(1963) *Cancer Res.*, **23**, 1474.
REES, E. D. AND HUGGINS, C.—(1960) *Cancer Res.*, **20**, 963.
REID, E.—(1964) *Br. J. Cancer*, **18**, 179.
SHONK, C. E., ARISON, R. N., KOVEN, B. J., MAJIMA, H. AND BOXER, G. E.—(1965) *Cancer Res.*, **25**, 206.
SHONK, C. E. AND BOXER, G. E.—(1964) *Cancer Res.*, **24**, 709.
SHONK, C. E., MORRIS, H. P. AND BOXER, G. E.—(1965) *Cancer Res.*, **25**, 671.
SMITH, J. A. AND KING, R. J. B.—(1966) *J. Endocr.*, **35** (in press).
STEDMAN, E. and STEDMAN, E.—(1944) *Nature, Lond.*, **153**, 499.
VILLEE, C. A. AND HAGERMAN, D. D.—(1958) *J. biol. Chem.*, **233**, 42.
WANG, T. Y.—(1965) *Proc. natn. Acad. Sci., U.S.A.*, **54**, 800.
WEBER, G. AND MORRIS, H. P.—(1963) *Cancer Res.*, **23**, 987.
WHITAKER, B. L.—(1961) *Br. J. Cancer*, **15**, 868.
YOUNG, S., COWAN, D. M. AND SUTHERLAND, L. E.—(1963) *J. Path. Bact.*, **85**, 331.